# Hypoxia Promotes Immune Evasion by Triggering β-Glucan Masking on the Candida albicans Cell Surface via Mitochondrial and cAMP-Protein Kinase A Signaling

**DOI:** 10.1128/mBio.01318-18

**Published:** 2018-11-06

**Authors:** Arnab Pradhan, Gabriela M. Avelar, Judith M. Bain, Delma S. Childers, Daniel E. Larcombe, Mihai G. Netea, Elena Shekhova, Carol A. Munro, Gordon D. Brown, Lars P. Erwig, Neil A. R. Gow, Alistair J. P. Brown

**Affiliations:** a Medical Research Council Centre for Medical Mycology, Institute of Medical Sciences, University of Aberdeen, Aberdeen, United Kingdom; b Department of Internal Medicine and Radboud Center for Infectious Diseases, Radboud University Medical Center, Nijmegen, Netherlands; c GlaxoSmithKline, Immunoinflammation Therapy Area, Stevenage, United Kingdom; Duke University Medical Center

**Keywords:** hypoxia, *Candida albicans*, cell wall, β-glucan masking, mitochondrial signaling, cAMP-protein kinase A signaling

## Abstract

Organisms must adapt to changes in oxygen tension if they are to exploit the energetic benefits of reducing oxygen while minimizing the potentially damaging effects of oxidation. Consequently, organisms in all eukaryotic kingdoms display robust adaptation to hypoxia (low oxygen levels). This is particularly important for fungal pathogens that colonize hypoxic niches in the host. We show that adaptation to hypoxia in the major fungal pathogen of humans Candida albicans includes changes in cell wall structure and reduced exposure, at the cell surface, of β-glucan, a key pathogen-associated molecular pattern (PAMP). This leads to reduced phagocytosis by murine bone marrow-derived macrophages and decreased production of IL-10, RANTES, and TNF-α by peripheral blood mononuclear cells, suggesting that hypoxia-induced β-glucan masking has a significant effect upon C. albicans-host interactions. We show that hypoxia-induced β-glucan masking is dependent upon both mitochondrial and cAMP-protein kinase A (PKA) signaling. The decrease in β-glucan exposure is blocked by mutations that affect mitochondrial functionality (*goa1*Δ and *upc2*Δ) or that decrease production of hydrogen peroxide in the inner membrane space (*sod1*Δ). Furthermore, β-glucan masking is enhanced by mutations that elevate mitochondrial reactive oxygen species (*aox1*Δ). The β-glucan masking defects displayed by *goa1*Δ and *upc2*Δ cells are suppressed by exogenous dibutyryl-cAMP. Also, mutations that inactivate cAMP synthesis (*cyr1*Δ) or PKA (*tpk1*Δ *tpk2*Δ) block the masking phenotype. Our data suggest that C. albicans responds to hypoxic niches by inducing β-glucan masking via a mitochondrial cAMP-PKA signaling pathway, thereby modulating local immune responses and promoting fungal colonization.

## Abstract (precis)

Animal, plant, and fungal cells occupy environments that impose changes in oxygen tension. Consequently, many species have evolved mechanisms that permit robust adaptation to these changes. The fungal pathogen Candida albicans can colonize hypoxic (low oxygen) niches in its human host, such as the lower gastrointestinal tract and inflamed tissues, but to colonize its host, the fungus must also evade local immune defenses. We reveal, for the first time, a defined link between hypoxic adaptation and immune evasion in C. albicans. As this pathogen adapts to hypoxia, it undergoes changes in cell wall structure that include masking of β-glucan at its cell surface, and it becomes better able to evade phagocytosis by innate immune cells. We also define the signaling mechanisms that mediate hypoxia-induced β-glucan masking, showing that they are dependent on mitochondrial signaling and the cAMP-protein kinase pathway. Therefore, hypoxia appears to trigger immune evasion in this fungal pathogen.

## INTRODUCTION

The relationship between an opportunistic pathogen and its human host is strongly influenced by the immune status of the host and the ability of the pathogen to evade immune detection and clearance. This is particularly evident for major fungal pathogens such as Candida albicans, Aspergillus fumigatus, and Cryptococcus neoformans which are contained or cleared by most healthy individuals but which can cause life-threatening disease in immunocompromised individuals, killing more than a million people worldwide each year (1).

In immunocompetent individuals, potent innate immune defenses provide a first line of defense against these pathogenic fungi once they have penetrated external physical barriers. Myeloid cells express an array of pattern recognition receptors (PRRs) that recognize fungal cells by interacting with specific pathogen-associated molecular patterns (PAMPs), some of which lie on the fungal cell surface (2, 3). The formation of an immunological synapse between a PRR and its cognate PAMP triggers signaling events in the myeloid cell that promote the phagocytosis and killing of the fungal cell and the activation of downstream immunological effectors (4, 5).

Meanwhile, the fungal pathogen attempts to evade and resist these immunological defenses. A. fumigatus expresses the RodA hydrophobin on the surfaces of spores to mask the PAMPs melanin and β-glucan, which would otherwise be detected by the phagocytic PRRs Dectin-1, Dectin-2, and MelLec (6). C. neoformans attempts to evade immune detection by enveloping itself in a polysaccharide capsule to mask β-glucan in its cell wall (7). Similarly, C. albicans modulates PAMP exposure on its cell surface in response to host-mediated and environmental signals (8–11). The degree of β-glucan exposure on the surfaces of C. albicans cells changes during the course of systemic infection (8), and C. albicans appears to actively modify β-glucan exposure at its surface. For example, the relatively low ambient pHs associated with vulvovaginal niches have been reported to trigger elevated β-glucan exposure, leading to enhanced innate recognition of C. albicans cells by macrophages and neutrophils (10). In contrast, host-derived lactate activates β-glucan masking via a noncanonical signaling pathway involving the lactate receptor Gpr1 and the transcription factor Crz1, and this leads to reduced phagocytic recognition and attenuated cytokine responses (9).

Further observations in mice and humans reinforce the importance of the PAMP β-glucan for the immune recognition of C. albicans. In humans, a Dectin-1 polymorphism that truncates this β-glucan receptor has been associated with aberrant cytokine responses to C. albicans and susceptibility to recurrent vulvovaginitis (12). In mice, the inactivation of Dectin-1 attenuates inflammatory responses to C. albicans and permits fungal proliferation in models of systemic, gastrointestinal, and mucosal infection (13–16). However, the degree to which Dectin-1 defects affect host immunity depends on the genetic background of the host and the adaptation of C. albicans *in vivo* (15, 17).

Once a C. albicans cell has been recognized and phagocytosed, the phagocyte attempts to kill the pathogen by launching a chemical assault upon the phagosome contents, which includes a burst of reactive oxygen, nitrogen, and other species (18). Certain combinations of stress appear to promote the killing of C. albicans cells (19). Nevertheless, the fungus attempts to resist killing by mounting robust oxidative, nitrosative stress responses that promote fungal survival (20–24).

Hypoxia (low oxygen) represents an additional stress that fungal pathogens are exposed to in the host (25). C. albicans displays a robust response to hypoxia (26–29), and consequently, this fungus is able to colonize hypoxic niches such as the gastrointestinal tract (30, 31), as well as aerobic niches such as the skin and mucosa. C. albicans cells appear to induce both short- and long-term transcriptional responses to hypoxia. Sellam and coworkers identified Sit4, Ccr4, and Sko1 as potential regulators of the short-term response, which includes the induction of the transcription factors Tye7 and Upc2 (29). C. albicans *sit4*, *ccr4*, and *sko1* mutants display hypoxic transcriptional signatures even under normoxic conditions, presumably because these mutations compromise mitochondrial functionality and oxygen utilization (29). The long-term response to hypoxia involves the upregulation of several pathways: glycolysis via Tye7 (32, 33), unsaturated fatty acid metabolism via Efg1 (26, 27), and sterol biosynthesis via Upc2 (34, 35).

We reasoned that, as low oxygen levels represent a significant input signal for fungal cells within certain host niches (25), and as hypoxia affects the expression of cell wall genes and proteins in C. albicans (26, 36), hypoxia might affect β-glucan exposure at the cell surface. Here we show that hypoxia induces β-glucan masking in C. albicans, we identify key signaling pathways that mediate hypoxia-induced β-glucan masking, and we demonstrate that hypoxia-induced β-glucan masking attenuates phagocytic recognition, uptake, and cytokine responses. This phenotype is likely to be important in the context of fungal immune detection and clearance during infection.

## RESULTS

### Hypoxia induces β-glucan masking at the C. albicans cell surface.

**FIG 1:**
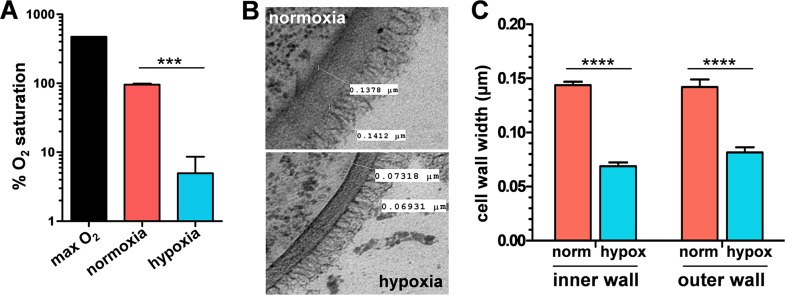
Hypoxia affects the architecture of the C. albicans cell wall. (A) Oxygen levels under the normoxic (pink) and hypoxic (blue) growth conditions used in this study. Means and standard deviations from three independent replicate experiments are shown. (B) Transmission electron micrographs of the cell walls of wild-type C. albicans cells (SC5314; see Table S1 in the supplemental material) grown under these normoxic and hypoxic conditions. (C) Quantification of the thickness of the inner and outer layers of the C. albicans cell wall using ImageJ from TEM images of SC5314 cells such as those shown in panel B. Means and standard deviations from images of cells (*n* = >30) from three independent replicate experiments are shown. The data were analyzed using ANOVA with Tukey’s multiple-comparison test and are indicated by asterisks as follows: *, *P* ≤ 0.05; **, *P* ≤ 0.01; ***, *P* ≤ 0.001; ****, *P* ≤ 0.0001.

To test the impact of hypoxia on the C. albicans cell wall, we grew cells under analogous conditions to those used to examine lactate-induced β-glucan masking (9). Wild-type cells (SC5314; see Table S1 in the supplemental material) were grown in minimal media under normoxic conditions and transferred to hypoxic conditions for five hours, and then cells were harvested for analysis while still in exponential growth phase (Materials and Methods). Dissolved oxygen levels were approximately 1% of the maximum levels under these conditions (Fig. 1A). Transmission electron microscopy (TEM) revealed that hypoxia affects the architecture of the C. albicans cell wall (Fig. 1B). Quantification of these TEM images revealed significant differences between hypoxic cells and their normoxic controls with respect to the thickness of the inner glucan-chitin and outer mannan layers of their cell walls. Hypoxic cells had thinner cell walls. This is consistent with the observations that hypoxia leads to changes in cell wall gene expression and the cell wall proteome (26, 36).

10.1128/mBio.01318-18.1 TABLE S1 C. albicans strains used in this study. Download Table S1, PDF file, 0.1 MB. Copyright © 2018 Pradhan et al. 2018 Pradhan et al. This content is distributed under the terms of the Creative Commons Attribution 4.0 International license.

**FIG 2:**
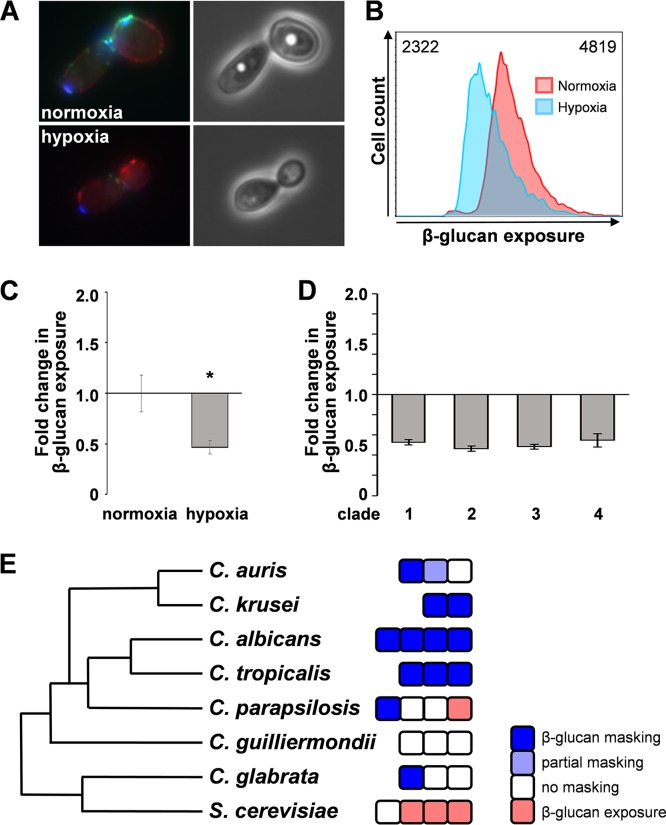
Hypoxia induces β-glucan masking in C. albicans. (A) Fluorescence microscopy of β-glucan exposure on C. albicans wild-type cells (SC5314: Table S1) grown under normoxic or hypoxic conditions and stained for exposed β-glucan (Fc-dectin-1; green), mannan (concanavalin A; red), and chitin (wheat germ agglutinin; blue). (B) Analysis of β-glucan exposure on C. albicans SC5314 cells grown under normoxic or hypoxic conditions by Fc-dectin-1 staining and flow cytometry. The median fluorescence intensity (MFI) for each population is indicated. (C) The fold change in β-glucan exposure for C. albicans SC5314 cells grown under hypoxic conditions was calculated relative to the values for control normoxic cells. Means and standard deviations from three independent replicate experiments are shown, and the data were analyzed using ANOVA with Tukey’s multiple-comparison test: *, *P* ≤ 0.05. (D) Quantification of hypoxia-induced β-glucan masking in C. albicans clinical isolates from four major clades: clade 1, SC5314; clade 2, IHEM16614; clade 3, J990102; clade 4, AM2005/0377 (Table S1). (E) Analysis of hypoxia-induced β-glucan masking in other pathogenic *Candida* species and in S. cerevisiae. Each box represents a different isolate (Table S1) (the data for C. albicans were taken from panel D). Masking was defined as a change in β-glucan exposure to <0.6 fold change (dark blue); partial masking was defined as a decrease in β-glucan exposure to between 0.6- and 0.8-fold change (light blue); no masking was defined as a change in β-glucan exposure between 0.8- and 1.2-old change decrease (white); β-glucan exposure was defined as an increase in β-glucan exposure to >1.4-fold change (pink).

We then tested whether hypoxia affects the degree of exposure of the major PAMP β-glucan at the C. albicans cell surface. First, β-glucan exposure on hypoxic and normoxic cells was examined by microscopy. Cells growing exponentially under normoxic and hypoxic conditions were harvested and fixed, and the exposed β-glucan was stained with Fc-Dectin-1 (Fig. 2A). The cells were also stained with wheat germ agglutinin (for chitin) and concanavalin A (for mannan). Hypoxic cells displayed less Fc-Dectin-1 staining than the control normoxic cells. This was quantified by flow cytometry (Fig. 2B), and the change in β-glucan exposure was then expressed in terms of the fold change in median fluorescence intensity (MFI) for the hypoxic cell population compared to the control normoxic population (Fig. 2C). Hypoxic cells displayed a significant decrease in MFI, indicating that hypoxia induces β-glucan masking in C. albicans.

Hypoxia-induced β-glucan masking was observed reproducibly in representative clinical isolates from four major epidemiological clades of C. albicans (Fig. 2D), indicating that this phenotype is not specific to clade 1 (SC5314). We also observed hypoxia-induced β-glucan masking in some other pathogenic *Candida* species, notably in Candida tropicalis and Candida krusei. However, most Candida glabrata, Candida guilliermondii, and Candida parapsilosis isolates did not display masking, and one of the Candida parapsilosis isolates we tested even displayed β-glucan exposure in response to hypoxia. The lack of a consistent β-glucan masking phenotype in these C. parapsilosis isolates might relate to this species’ apparent habitation of diverse environmental niches as well as being a skin commensal (37). There was no clear correlation between the hypoxia-induced β-glucan masking phenotype and phylogenetic relatedness (Fig. 2E).

### Hypoxia-induced β-glucan masking is not dependent on the pathway that mediates lactate-induced β-glucan masking.

**FIG 3:**
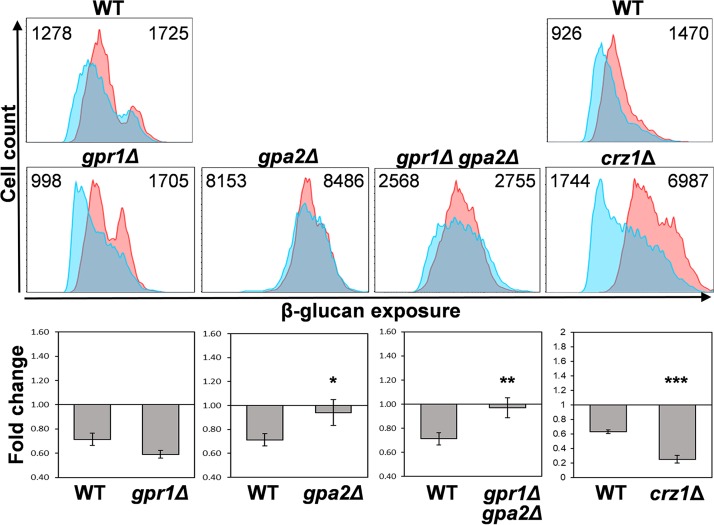
Hypoxia-induced β-glucan masking is not dependent on Gpr1 or Crz1. Analysis of β-glucan exposure on C. albicans mutants by flow cytometry of Fc-dectin-1-stained cells grown under normoxic (pink) or hypoxic conditions (cyan). The median fluorescence intensity (MFI) for each population is shown at the top right and left of each panel, respectively: WT, wild type, SC5314; *gpr1*Δ, LR2; *gpa2*Δ, NM6; *gpr1*Δ *gpa2*Δ, NM23; *crz11*Δ, DSY2195 (Table S1). The wild-type control for each experiment is shown above the mutants examined in that same experiment. The *gpr1*Δ and *gpa2*Δ mutants (middle panels) were compared together in the same experiment with the wild-type control (upper left panel), whereas the *crz1*Δ mutant (middle panel) was compared with wild-type cells in a different experiment (upper right panel). The fold changes in β-glucan exposure for each strain (lower panels) were calculated by dividing the MFI under hypoxic conditions by the MFI for the corresponding normoxic cells. Means and standard deviations from three independent replicate experiments are shown, and the data were analyzed using ANOVA with Tukey’s multiple-comparison test: *, *P* ≤ 0.05; **, *P* ≤ 0.01; ***, *P* ≤ 0.001.

Previously we showed that lactate-induced β-glucan masking is mediated by a noncanonical signaling pathway that involves lactate receptor Gpr1 and transcription factor Crz1 (9). Therefore, we tested whether hypoxia-induced β-glucan masking is mediated by the same pathway. β-Glucan masking was quantified by flow cytometry in C. albicans mutants that lack Gpr1, its Gα protein Gpa2, or Crz1 (Fig. 3) (Table S1). The deletion of *CRZ1* affected β-glucan exposure: both hypoxic and normoxic *crz1*Δ cell populations displayed elevated levels of exposure relative to wild-type control cells. However, the loss of Crz1 did not block hypoxia-induced β-glucan masking. Also, *gpr1*Δ cells retained the β-glucan masking phenotype (Fig. 3). Therefore, neither Gpr1 nor Crz1 is required for hypoxia-induced β-glucan masking, indicating that the different cellular inputs, lactate and hypoxia, trigger β-glucan masking via different signaling pathways. Interestingly, the Gα protein Gpa2 is required for hypoxia-induced β-glucan masking (Fig. 3).

### Hypoxia-induced β-glucan masking is not dependent on key morphogenetic or stress regulators.

Key morphogenetic regulators such as Efg1, stress regulators such as Hog1, and the cell integrity signaling pathway are known to influence cell wall gene expression and cell wall structure in C. albicans (26, 38–44). Furthermore, Efg1 contributes to the regulation of the hypoxic response in C. albicans (26, 27), and Hog1 orthologues contribute to hypoxic responses in Saccharomyces cerevisiae and human cells (45, 46). Therefore, we tested whether these and other related regulators are required for hypoxia-induced β-glucan masking.

**FIG 4:**
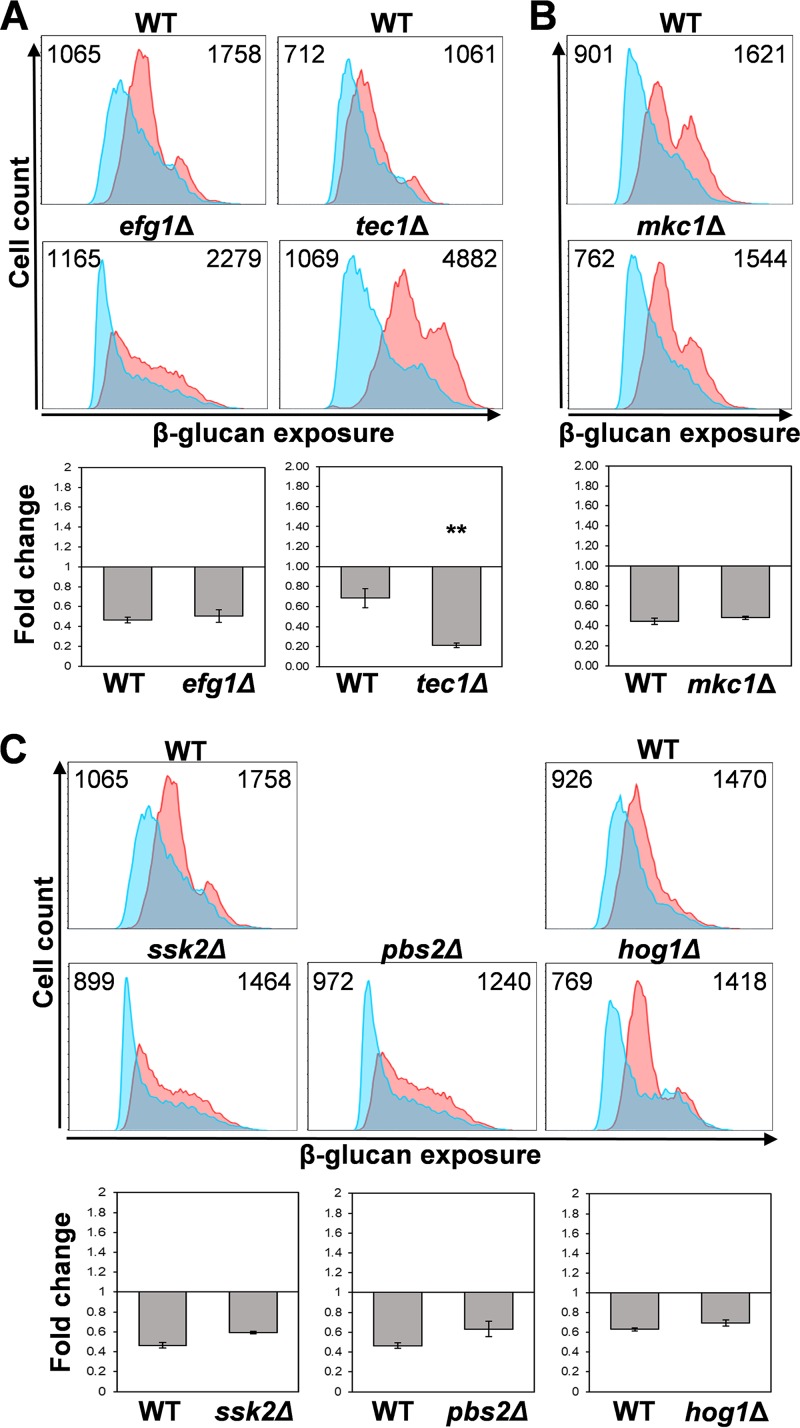
Hypoxia-induced β-glucan masking is not dependent on key regulators of morphogenesis, cell integrity, or stress adaptation. Analysis of β-glucan exposure on C. albicans mutants by flow cytometry of Fc-dectin-1-stained cells (upper panels) grown under normoxic (pink) or hypoxic conditions (cyan). Median fluorescence intensities (MFIs) for hypoxic and normoxic cell populations are shown (top right and left of each panel, respectively). The corresponding wild-type control is shown above each mutant. The fold change in β-glucan exposure (lower panels) for each strain was calculated by dividing the MFI under hypoxic conditions by the MFI for the control normoxic cells. Means and standard deviations from three independent replicate experiments are shown, and the data were analyzed using ANOVA with Tukey’s multiple-comparison test: *, *P* ≤ 0.05; **, *P* ≤ 0.01. (A) Select morphogenetic mutants are shown in WT (wild type) (SC5314) and *efg1*Δ (HLC52) and *tec1*Δ (CaAS18) mutants (Table S1). Additional mutants are shown in Fig. S1. (B) Cell integrity pathway in the WT (SN95) and *mkc1*Δ (CaLC700) cells. (C) Stress-activated protein kinase pathway in WT (SC5314) and *ssk2*Δ (JC482), *pbs2*Δ (JC74), and *hog1*Δ (JC50) mutants. The *efg1*Δ, *ssk1*Δ, and *pbs2*Δ strains were analyzed in the same experiment against the same wild-type control.

Hypoxia-induced β-glucan masking was retained in *efg1*Δ cells (Fig. 4A). Furthermore, the phenotype was maintained in *tec1*Δ, *cph1*Δ, and *bcr1*Δ cells (Fig. 4A; see also Fig. S1 in the supplemental material). We note that the basal levels of β-glucan exposure were perturbed in *tec1*Δ and *efg1*Δ cells, presumably because these mutations perturb the yeast cell wall (41, 47). Nevertheless, the *tec1*Δ and *efg1*Δ mutants still displayed β-glucan masking in response to hypoxia (Fig. 4A and Fig. S1). Therefore, the morphogenetic regulators Efg1, Tec1, Cph1, and Bcr1 are not required for hypoxia-induced β-glucan masking.

10.1128/mBio.01318-18.2 FIG S1 Hypoxia-induced β-glucan masking in specific C. albicans mutants. Analysis of β-glucan masking by C. albicans mutants by Fc-dectin-1 staining of cells grown under normoxic (pink) or hypoxic conditions (cyan) (upper panels). Median fluorescence intensities (MFIs) for hypoxic and normoxic cell populations are shown. The corresponding wild-type control is shown above each mutant or set of mutants. Fold changes in β-glucan exposure (lower panels) represent the MFI under hypoxic conditions divided by the MFI for the corresponding control normoxic cells. Means and standard deviations from three independent replicate experiments are shown, and the data were analyzd using ANOVA with Tukey’s multiple-comparison test: *, *P* ≤ 0.05; **, *P* ≤ 0.01; ***, *P* ≤ 0.001. (A) Morphogenetic mutants. WT, wild type, SC5314; *cph1*Δ, JKC19; *efg1*Δ, HLC52; *bcr1*Δ, CNJ702; *tec1*Δ, CaAS18 (Table S1). (B) Mutants affecting mitochondrial functionality. WT, wild type, DAY152; *pop2*Δ, YCAT51; *ccr4Δ*, YCAT39. The data for the *efg1* Δ, *tec1*Δ, and *ccr4*Δ mutants also appear in Fig. 4 and 6. Download FIG S1, PDF file, 0.2 MB. Copyright © 2018 Pradhan et al. 2018 Pradhan et al. This content is distributed under the terms of the Creative Commons Attribution 4.0 International license.

The Mkc1 MAP kinase is critical for signaling via the cell integrity pathway (39, 40). However, hypoxia-induced β-glucan masking was not perturbed in *mkc1*Δ cells (Fig. 4B), indicating that this masking is not dependent on the cell integrity pathway.

C. albicans cells lacking the Hog1 stress-activated protein kinase, its MAP kinase kinase Pbs2, or its MAP kinase kinase kinase Ssk2 (48, 49) retained hypoxia-induced β-glucan masking (Fig. 4C). Therefore, despite the fact that Hog1 signaling contributes to cell wall remodelling in C. albicans and to hypoxic responses in other eukaryotes (43, 45, 46), this signaling pathway is not essential for hypoxia-induced β-glucan masking in C. albicans.

### Hypoxia-induced β-glucan masking is dependent on cAMP-protein kinase A signaling.

**FIG 5:**
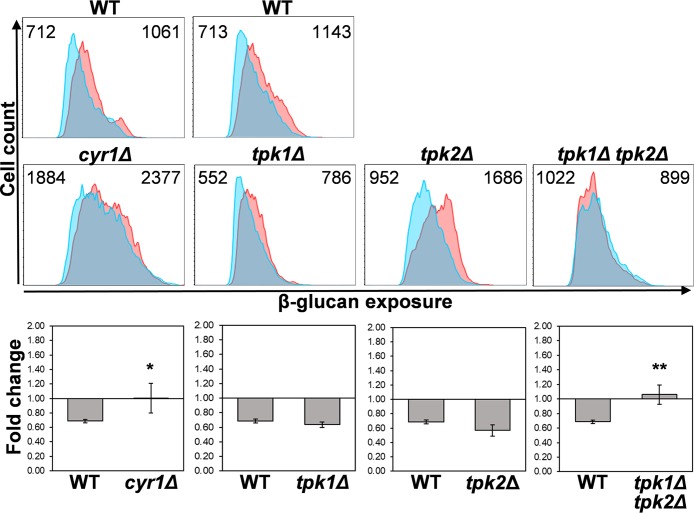
Hypoxia-induced β-glucan masking is dependent on cAMP-PKA signaling. Cytometric analysis of β-glucan exposure on C. albicans cAMP-PKA mutants by Fc-dectin-1 staining of cells grown under normoxic (pink) or hypoxic conditions (cyan) (upper panels). Median fluorescence intensities (MFIs) for hypoxic and normoxic cell populations are shown. The corresponding wild-type control is shown above each mutant: WT, wild type (SN152) and *cyr1*Δ (CR323), *tpk1*Δ, *tpk2Δ*, and *tpk1*Δ *tpk2*Δ mutants (Table S1). The fold change in β-glucan exposure (lower panels) for each strain was calculated by dividing the MFI under hypoxic conditions by the MFI for the corresponding control normoxic cells. Means and standard deviations from three independent replicate experiments are shown, and the data were analyzed using ANOVA with Tukey’s multiple-comparison test: *, *P* ≤ 0.05; **, *P* ≤ 0.01.

The cAMP-protein kinase A (PKA) pathway plays important roles in yeast-hypha morphogenesis, stress adaptation, and cell wall integrity in C. albicans (50–56). Therefore, we tested whether cAMP-PKA signaling is required for hypoxia-induced β-glucan masking. First, we examined C. albicans *cyr1*Δ cells which lack adenylyl cyclase (51), and then we tested *tpk1*Δ and *tpk2*Δ mutants in which one or both types of PKA catalytic subunit have been inactivated (56). Significantly, hypoxia-induced β-glucan masking was blocked in *cyr1*Δ cells and also in the *tpk1*Δ *tpk2*Δ double mutant, which lacks any PKA activity (Fig. 5). This indicates that cAMP-PKA signaling is critical for this phenotype. Interestingly, the phenotype was retained in the single *tpk1*Δ and *tpk2*Δ mutants (Fig. 5), showing that each type of catalytic subunit of PKA is capable of mediating hypoxia-induced β-glucan masking.

### Mutations that attenuate mitochondrial respiration affect hypoxia-induced β-glucan masking.

Next, we investigated possible mechanisms by which hypoxia might activate cAMP-PKA signaling. Structural and functional alterations in mitochondrial respiratory chain complexes are known to increase superoxide levels. Mitochondrial reactive oxygen species (ROS), such as superoxide, become elevated under hypoxic conditions, and these mitochondrial ROS are thought to contribute to hypoxic signaling (57, 58). Therefore, we examined whether mutations that affect mitochondrial functionality in C. albicans perturb hypoxia-induced β-glucan masking.

**FIG 6:**
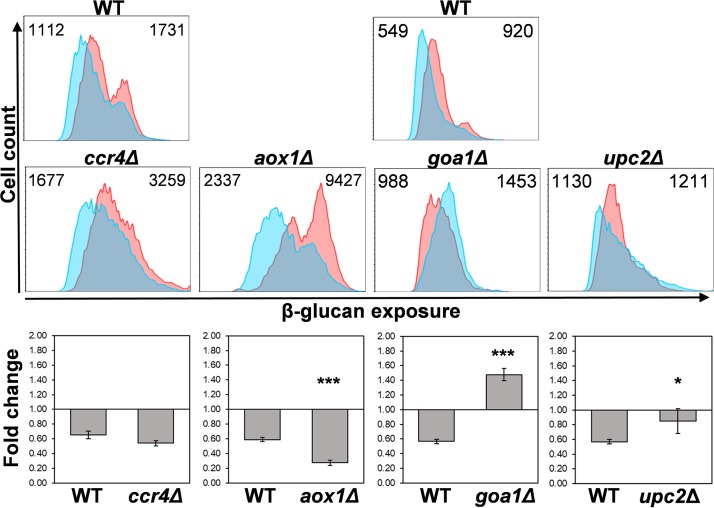
Mutations that perturb mitochondrial functionality affect hypoxia-induced β-glucan masking. Quantification of β-glucan exposure on C. albicans mutants by Fc-dectin-1 staining and flow cytometry of cells grown under normoxic (pink) or hypoxic conditions (cyan) (upper panels): WT, wild type (DAY185), *ccr4*Δ (YCAT39), *aox1*Δ (WH324), *goa1Δ* (GOA31), *upc2*Δ (UPC2M4A) (Table S1). Additional mutants are shown in Fig. S1. The cytometry data for the corresponding wild-type control is shown above each set of mutants analyzed in the same experiment. Median fluorescence intensities (MFIs) for hypoxic and normoxic cell populations are shown. The fold change in β-glucan exposure (lower panels) for each strain represents the MFI under hypoxic conditions divided by the MFI for the corresponding normoxic cells. Means and standard deviations from three independent replicate experiments are shown, and the data were analyzed using ANOVA with Tukey’s multiple-comparison test: *, *P* ≤ 0.05; **, *P* ≤ 0.01; ***, *P* ≤ 0.001.

We examined *ccr4*Δ and *pop2*Δ mutants because the inactivation of this Ccr4-Pop2 mRNA deadenylase (Table S1), which regulates mRNA decay and translation, has been reported to affect mitochondrial phospholipid homeostasis and to confer sensitivity to cell wall stressors in C. albicans (59). Presumably, these pleiotropic effects account for the relatively high levels of β-glucan exposure observed for the *ccr4*Δ and *pop2*Δ cells under basal, normoxic conditions (Fig. 6 and Fig. S1). Nevertheless, hypoxia-induced β-glucan masking was retained in *ccr4*Δ and *pop2*Δ strains and was even enhanced in *pop2*Δ cells (Fig. 6 and Fig. S1).

*AOX1A* and *AOX1B* are nonsynonymous alleles which encode alternative oxidases that protect the C. albicans mitochondrion against oxidative damage (60–62), as do their homologues in other fungi and plants (63, 64). We observed that, like *pop2*Δ cells, a C. albicans *aox1A*Δ/*aox1B*Δ (hereafter *aox1*Δ) mutant that lacks both isoforms of Aox1, display significantly enhanced β-glucan masking in response to hypoxia compared to the wild-type control (Fig. 6). Normoxic *aox1*Δ cells displayed high basal levels of β-glucan exposure, relative to the wild-type control (Fig. 6), potentially because of abnormally high levels of mitochondrial ROS (60–64).

We then determined whether Goa1 or Upc2 influences the phenotype because both of these proteins have been implicated in mitochondrial functionality. The exact biochemical role of Goa1 remains obscure, but a *GOA1* gene deletion confers oxidative stress sensitivity and results in the loss of mitochondrial membrane potential (65). In addition to its roles in the regulation of ergosterol biosynthesis, the transcription factor Upc2 regulates the expression of essential mitochondrial chaperones, mediates lipid homeostasis, and contributes to the hypoxic response in C. albicans (33, 35, 66–68). Hypoxia-induced β-glucan masking was blocked in C. albicans *goa1*Δ and *upc2*Δ mutants (Fig. 6 and Table S1). This further implicates mitochondrial signaling in activating this phenotype.

**FIG 7:**
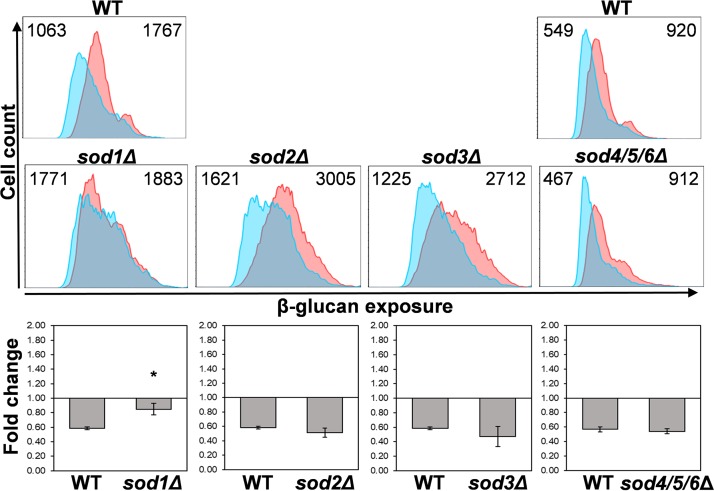
Sod1 is required for hypoxia-induced β-glucan masking. C. albicans superoxide dismutase mutants were grown under normoxic (pink) or hypoxic conditions (cyan), stained with Fc-dectin-1, and analyzed by flow cytometry to examine their β-glucan exposure (upper panels). Median fluorescence intensities (MFIs) for hypoxic and normoxic cell populations are shown. The corresponding wild-type control is shown above each set of mutants, the triple *sod4-6*Δ mutant having been analyzed in a separate experiment from the other *sod*Δ mutants: WT, SC5314; *sod1*Δ, CA-IF003; *sod2*Δ, CA-IF007; *sod3*Δ, CA-IF011 single mutants; *sod4/5/6*Δ triple mutant, CA-IF070 (Table S1). The fold change in β-glucan exposure (lower panels) for each strain represents the MFI under hypoxic conditions relative to the MFI for the corresponding normoxic control. Means and standard deviations from three independent replicate experiments are shown, and the data were analyzed using ANOVA with Tukey’s multiple-comparison test: *, *P* ≤ 0.05.

Changes in mitochondrial superoxide levels have been suggested to contribute to hypoxic signaling (57, 58). Therefore, we tested whether specific C. albicans superoxide dismutases affect hypoxia-induced β-glucan masking. C. albicans expresses six superoxide dismutases: Sod1 is localized to the intermembrane space and mitochondrial matrix, Sod2 to the mitochondrial matrix and Sod3 to the cytoplasm, whereas Sod4 to 5 are secreted (21, 62, 69–72). Mutants lacking Sod2, Sod3, Sod4, Sod5, and/or Sod6 displayed no obvious defects in hypoxia-induced β-glucan masking (Fig. 7). Although no masking defect was observed for *sod2*Δ and *sod3*Δ cells, they did display relatively high levels of β-glucan exposure under basal normoxic conditions (Fig. 7). The basis for this is not clear, but it might be related to the perturbation of intracellular ROS under normal growth conditions. However, C. albicans *sod1*Δ cells, which lack the only superoxide dismutase that is localized to the intermembrane space (62), displayed a significant reduction in hypoxia-induced β-glucan masking (Fig. 7). This is consistent with the view that ROS signaling in the mitochondrial intermembrane space is involved in activating β-glucan masking.

**FIG 8:**
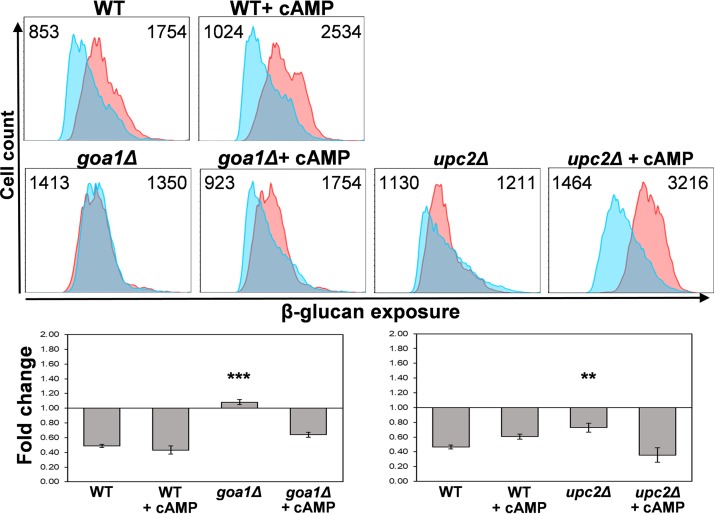
Exogenous dibutyryl-cAMP suppresses the defects in hypoxia-induced β-glucan masking caused by mitochondrial mutants. C. albicans (wild type [SC5314] [Table S1]), *goa1*Δ (GOA31), and *upc2*Δ cells (UPC2M4A) were grown under normoxic (pink) or hypoxic conditions (cyan) for 5 h, as described above, with 0 or 5 mM dibutyryl-cAMP (cAMP). The cells were then stained with Fc-dectin-1 and analyzed by flow cytometry to quantify their β-glucan exposure (upper panels). Median fluorescence intensities (MFIs) are shown. The fold changes in β-glucan exposure are shown (lower panels): means and standard deviations from three independent replicate experiments are analyzed using ANOVA with Tukey’s multiple-comparison test: *, *P* ≤ 0.05; **, *P* ≤ 0.01; ***, *P* ≤ 0.001.

Both the cAMP-PKA pathway (Fig. 5) and mitochondrial signaling (Fig. 6) are required for hypoxia-induced β-glucan masking. To test whether the cAMP signaling might lie upstream or downstream of mitochondrial signaling, we asked whether exogenous cAMP can suppress the defect in masking observed for *goa1*Δ and *upc2*Δ cells. We used the membrane-permeable derivative, dibutyryl-cAMP (db-cAMP) for these experiments as described previously (73). Interestingly, exogenous db-cAMP suppressed the masking defect of *goa1*Δ and *upc2*Δ cells (Fig. 8), suggesting that mitochondrial signaling might lie upstream of the cAMP-PKA pathway.

### Hypoxia-induced β-glucan masking attenuates immune recognition and host responses.

The interactions of C. albicans with innate immune cells are affected when β-glucan masking is activated by lactate (9). In particular, lactate-induced β-glucan masking reduces neutrophil recruitment, decreases phagocytosis by macrophages, and attenuates cytokine responses. Therefore, we tested whether hypoxia-induced β-glucan masking exerts similar effects upon immune responses.

**FIG 9:**
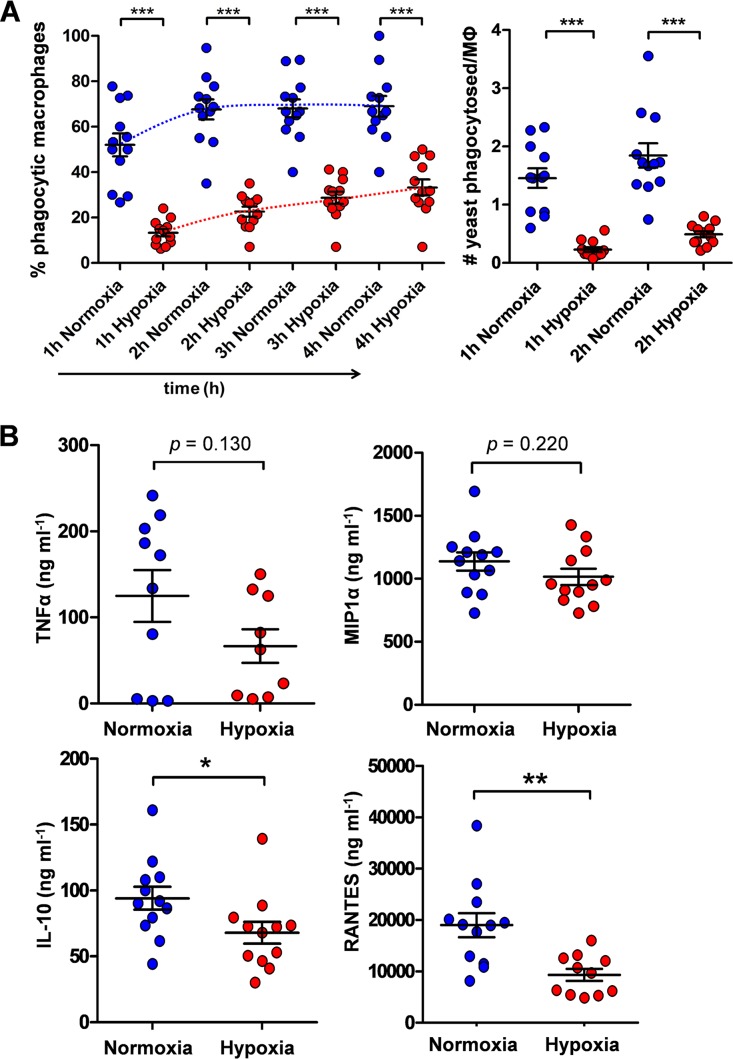
Growth under hypoxia attenuates immune responses against C. albicans. Wild-type C. albicans cells (SC5314 [Table S1]) were grown for 5 h under normoxic (blue) or hypoxic conditions (red) and fixed. (A) At *t* = 0, these C. albicans cells were mixed with murine bone marrow-derived macrophages (BMDMs) at a ratio of 3:1 (yeast cells/macrophages), and the host-fungus interactions monitored by time-lapse video microscopy. The proportion of BMDMs that had phagocytosed at least one C. albicans cell (percent phagocytic macrophages) was quantified at *t* = 1, 2, 3, and 4 h. Also, the number of C. albicans cells phagocytosed per BMDM were quantified at *t* = 1 and 2 h. (B) Duplicate samples of human PBMCs from 6 different individuals were mixed with the C. albicans cells (ratio of 5:1, yeast cells/PBMCs), and TNF-α, MIP-1α, IL-10, and RANTES levels were assayed after 24 h. These data were analyzed using ANOVA with Bonferroni’s *post hoc* test: *, *P* ≤ 0.05; **, *P* ≤ 0.01; ***, *P* ≤ 0.001.

We quantified the phagocytosis of wild-type C. albicans cells by primary murine bone marrow-derived macrophages (BMDMs) from time-lapse spinning disc video microscopy. Representative videos can be viewed in Movies S1 and S2 in the supplemental material. Over the 4-h period examined, significantly fewer macrophages phagocytosed the C. albicans cells that had undergone hypoxia-induced β-glucan masking compared with the unmasked control cells (Fig. 9A). Furthermore, these macrophages ingested fewer of the masked C. albicans cells than the unmasked cells (Fig. 9A). This was consistent with the view that hypoxia-induced β-glucan masking renders C. albicans cells less visible to phagocytes. It was not possible to monitor the fate of these C. albicans cells after phagocytosis because, for technical reasons, these cells were fixed (Materials and Methods).

10.1128/mBio.01318-18.3 MOVIE S1 Time-lapse video of BMDM interactions with normoxic C. albicans cells. Movies S1 and S2, which are representative of 12 movies in total (4 movies from 3 mice), show the first two hours of interactions between murine BMDMs and normoxic C. albicans interactions. Download Movie S1, AVI file, 18.8 MB. Copyright © 2018 Pradhan et al. 2018 Pradhan et al. This content is distributed under the terms of the Creative Commons Attribution 4.0 International license.

10.1128/mBio.01318-18.4 MOVIE S2 Time-lapse video of BMDM interactions with normoxic C. albicans cells. Movies S1 and S2, which are representative of 12 movies in total (4 movies from 3 mice), show the first two hours of interactions between murine BMDMs and normoxic C. albicans interactions. Download Movie S2, AVI file, 18.7 MB. Copyright © 2018 Pradhan et al. 2018 Pradhan et al. This content is distributed under the terms of the Creative Commons Attribution 4.0 International license.

We then quantified cytokine and chemokine responses for peripheral blood mononuclear cells (PBMCs) isolated from blood samples from healthy human volunteers. We observed a slight reduction in the levels of TNF-α and possibly MIP-1α induced by the masked C. albicans cells compared to the control unmasked cells, but these changes were not statistically significant (Fig. 9B). However, the masked cells induced significantly lower levels of the anti-inflammatory cytokine IL-10, and of the chemokine RANTES (Fig. 9B), which plays an important role in the homing and migration of effector and memory T cells. Taken together, the data clearly show that hypoxia-induced β-glucan masking affects immune responses to C. albicans cells.

## DISCUSSION

Oxygen levels can vary greatly in healthy tissues, but tissues can become hypoxic during infection, and oxygen levels approach zero in the lumen of the healthy lower gastrointestinal tract, permitting colonization by obligate anaerobes (25, 74–78). C. albicans displays robust adaptation to hypoxic environments, and consequently is able to colonize such niches (26–29, 31, 79, 80). As hypoxia has been shown to affect the expression of cell wall genes and proteins in C. albicans (26, 36), we hypothesized that this host input might affect cell wall architecture, and in particular, PAMP exposure at the C. albicans cell surface. We tested this and showed that hypoxia induces significant changes in the thickness of the inner glucan-chitin and outer mannan layers of the cell wall (Fig. 1) and that hypoxia also induces β-glucan masking (Fig. 2).

Previously we showed that host-derived lactate triggers β-glucan masking in C. albicans (9). We reasoned that, given the different nature of these host inputs, lactate and hypoxia might mediate β-glucan masking via different upstream regulators. As we predicted, hypoxia-induced β-glucan masking is not dependent on the lactate receptor Gpr1 (Fig. 3) (9). However, the Gpr1-associated G-alpha protein, Gpa2, does contribute to both lactate-induced (9) and hypoxia-induced β-glucan masking (Fig. 3). This implies that Gpa2 must also be regulated by Gpr1-independent mechanisms.

**FIG 10:**
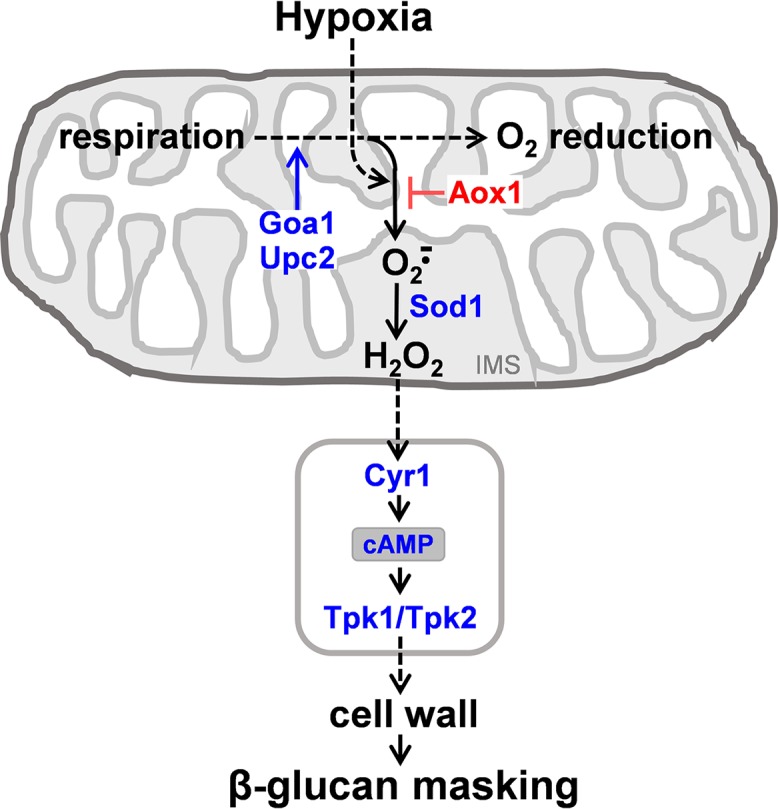
Mechanisms by which hypoxia induces β-glucan masking in C. albicans. Combining our observations with those of others, we propose the following working model. Hypoxia triggers an increase in the formation of mitochondrial superoxide by the respiratory apparatus (57, 58). Inactivating Goa1 or Upc2, which promote mitochondrial functionality, reduces overall respiration rates and hence mitochondrial ROS production. The alternative oxidase (Aox1) acts to limit mitochondrial ROS production (60–62) and therefore inactivating Aox1 enhances the signal. Superoxide dismutase within the mitochondrial inner membrane space (IMS) converts superoxide into diffusible hydrogen peroxide, which leads to the generation of a mitochondrial signal that transduces to the cytoplasm (see text). This possibly leads to the activation of adenylyl cyclase (Cyr1) and cAMP-PKA (Tpk1/2) signaling, which triggers remodelling of the cell wall and masking of cell surface β-glucan by mechanisms that remain to be elaborated.

Our data suggest that the hypoxic signal is mediated via the mitochondrion (Fig. 10), which would be consistent with data from fungal, plant, and mammalian systems (57, 58, 81–84). First, the inhibition of mitochondrial functionality in *C. albicans* (*goa1*, *upc2*) blocked hypoxia-induced β-glucan masking (Fig. 6).

Second, C. albicans cells that lack the mitochondrial alternative oxidase, Aox1, display enhanced hypoxia-induced β-glucan masking (Fig. 6). In C. albicans and other fungi and in plants, alternative oxidases such as Aox1 limit the superoxide generation in the respiratory chain, thereby protecting the mitochondrion against oxidative damage (60–64). Therefore, hypoxia-induced β-glucan masking is probably enhanced in response to the elevated superoxide levels in the mitochondrion of *aox1*Δ cells.

Third, the masking phenotype is blocked in *sod1*Δ cells, but not in C. albicans cells that lack any of the other superoxide dismutases (Fig. 7). Sod1 is the only superoxide dismutase that localizes to the mitochondrial intermembrane space (62). This is particularly significant because respiratory complex III releases superoxide into the mitochondrial intermembrane space (57, 85–87) and complex III is critical for hypoxic signaling (57, 58, 81, 84, 88, 89). Normally, in wild-type cells, Sod1 converts the charged, nondiffusible superoxide anion into the diffusible ROS, hydrogen peroxide. Thus, in *sod1*Δ cells, hydrogen peroxide production would be lowered in the mitochondrial intermembrane space.

Taken together, these data suggest that the transduction of the hypoxic signal depends on the generation of hydrogen peroxide in the mitochondrial intermembrane space (Fig. 10). Hydrogen peroxide is viewed as a candidate signaling molecule because of its relatively long half-life, its membrane permeability, and its ability to oxidize cysteines in target proteins (90, 91). Certainly, cysteine oxidative modifications have been shown to regulate the activities of key proteins in involved in gene expression, metabolism, cell differentiation, and growth (92, 93). Indeed, perturbations of cytochrome *c* oxidase or cytochrome *c* itself could conceivably trigger downstream signaling events, as the inactivation of these proteins has been shown to affect signaling in other systems (94, 95). Therefore, we suggest that hydrogen peroxide might trigger downstream signaling events in hypoxic C. albicans cells.

The downstream transduction of the mitochondrial signal generated is not dependent on Hog1 signaling or Efg1 (Fig. 4), which has been implicated previously in transcriptional responses to hypoxia (26, 29, 45, 46). Instead, hypoxic signal transduction is dependent on the cAMP-PKA pathway (Fig. 5, 8, and 10). It is conceivable that, in some way, the mitochondrial and cAMP-PKA pathways act in parallel. However, hypoxia-induced glucan β-masking is dependent upon both signaling modules.

The involvement of cAMP-PKA signaling in β-glucan masking is entirely consistent with previous work showing that this pathway influences cell wall gene expression and integrity (52, 55, 56). Furthermore, our observations reinforce the view that the mitochondrion and adenylyl cyclase control virulence phenotypes in C. albicans (96). The mechanisms by which β-glucan masking is achieved at the cell surface remain obscure and are under investigation. However, transcript profiling studies of adenylyl cyclase and PKA mutants suggest that this pathway modulates the synthesis and assembly of cell wall mannoproteins and mannan (*ALS1*, *ALS2*, *ALS4*, *CCW14*, *CSP37*, *ECM4*, *KTR1*, *SCW10*, *WSC1*), glucan (*KRE6*, *KRE9*, *PHR2*) as well as chitin (*CHS7*, *CHT3*) (52, 55), potentially providing clues as to these mechanisms.

Changes in β-glucan exposure on C. albicans cells have been observed *in vivo*, during systemic infection (8), and *in vitro* in response to changes in ambient pH or host-derived lactate (9, 10). It is well-known that β-glucan recognition by Dectin-1 plays a major role in fungal recognition by innate immune cells (4, 13–16, 97). It has been reported that C. albicans cells grown overnight under hypoxia with high carbon dioxide levels (5% CO_2_) display enhanced immune recognition (17). Here we report that exponentially growing C. albicans cells, which were exposed specifically to hypoxia before fixing, elicit attenuated immune responses (Fig. 9), like cells exposed to lactate (9). We reason that differences in CO_2_ concentration, cell morphology, and/or growth state might account for the different immunological outputs in these two studies. Certainly, high CO_2_ concentrations are known to affect C. albicans morphology and physiology (98), and C. albicans morphology affects innate immune responses (99, 100).

We observed different phagocytic responses for BMDMs toward hypoxic C. albicans cells compared to normoxic control cells. Hypoxic cells evaded phagocytic uptake despite numerous contacts between yeast cells and phagocytes during their dynamic interactions over the period examined (see Movies S1 and S2 in the supplemental material). This was presumably because of the reduced availability of fungal target sites for host cell Dectin-1 engagement. The impact of hypoxia upon innate immune responses against C. albicans cells was more subtle than for those previously observed for lactate exposure. Exposing the fungal cells to lactate led to significant reductions in the levels of TNF-α and MIP1α released by human macrophages (9). Hypoxia-grown C. albicans cells also elicited reduced levels of these cytokines compared to control normoxic cells, but these changes were less dramatic and not statistically significant (Fig. 9). However, statistically significant decreases in IL-10 and RANTES production were observed for hypoxic cells (Fig. 9), indicating that hypoxia does affect immune responses against C. albicans. These differences in cytokine responses between lactate- and hypoxia-treated C. albicans cells might relate to the different signaling mechanisms that are activated in response to these host inputs (above). No doubt these different signaling mechanisms drive subtly different patterns of cell wall remodelling, in addition to the common β-glucan masking phenotype we have described.

We argue that the effects of hypoxia on β-glucan masking by C. albicans and upon the innate immune responses against this pathogen will almost certainly have a significant impact upon host-fungus interactions during colonization and infection. This view is supported by the accompanying paper (101), which shows that oxygen deprivation enhances the successful colonization of host niches by C. albicans *in vivo*.

## MATERIALS AND METHODS

### Strains and growth conditions.

Strains are listed in Table S1 in the supplemental material. All C. albicans strains were grown overnight at 30°C and 200 rpm in minimal medium (GYNB [2% glucose, 0.65% yeast nitrogen base without amino acids, containing the appropriate supplements]) (102). On the day of an experiment, overnight cultures were diluted into fresh minimal medium to an OD_600_ of 0.2, and incubated at 30°C at 200 rpm for 5 h for analysis. Normoxic cells were grown with aeration, whereas hypoxic cells were grown in screw cap conical flasks under nitrogen. Dissolved O_2_ was measured using a Thermo Fisher Scientific Orion RDO probe 3M (087010MD).

### Microscopy.

For fluorescence microscopy, cells were fixed in 50 mM thimerosal (Sigma-Aldrich) and stained for β-glucan (1.5 µg/ml Fc-Dectin-1 plus anti-human IgG conjugated to Alexa Fluor 488; green), chitin (50 µg/ml wheat germ agglutinin conjugated to Alexa Fluor 350; blue), and mannan (25 µg/ml concanavalin A conjugated to Texas Red; red). All samples were examined by phase differential interference contrast (DIC) and fluorescence microscopy using a Zeiss Axioplan 2 microscope. Images were recorded digitally using the Openlab system (Openlab v 4.04: Improvision, Coventry, UK) with a Hamamatsu C4742- 95 digital camera (Hamamatsu Photonics, Hamamatsu, Japan).

High-pressure freeze substitution transmission electron microscopy on normoxic and hypoxic C. albicans cells was performed as described previously (103, 104), cutting ultrathin sections of 100 nm in thickness. Samples were imaged with a Philips CM10 transmission microscope (FEI, United Kingdom) equipped with a Gatan Bioscan 792 camera, and the images were recorded using a Digital Micrograph (Gatan, Abingdon Oxon, United Kingdom). The thicknesses of the inner chitin-glucan and outer mannan layers of the cell wall were measured by averaging >30 measurements for each cell (*n* > 30 cells) using ImageJ.

### β-Glucan exposure.

To assess the exposure of β-glucan on the C. albicans cell surface, strains were grown in YNB plus 2% glucose overnight and then grown in fresh medium for 5 h under hypoxic or normoxic conditions. These exponentially growing cells were fixed immediately with 50 mM thimerosal (Sigma-Aldrich, Dorset, UK) to capture the cell surface architecture. They were then stained for β-glucan exposure using Fc-Dectin-1 and anti-human IgG conjugated to Alexa Fluor 488, and their fluorescence was quantified using a BD Fortessa flow cytometer as described previously (9). The plots represent three biological replicate experiments, in each of which 10,000 events were acquired. Normoxic cells of the congenic wild-type control were used as a control for each run. As a secondary control, cells were treated as descrbied above but without the addition of Fc-Dectin-1. Median fluorescence intensities (MFI) were determined using FlowJo v. 10 software.

### Cytokine assays.

Cytokine assays on PBMCs were performed as described previously (9). Briefly, PBMCs were isolated from nonheparinized whole-blood samples (20 ml) collected from healthy donors using Ficoll-Paque centrifugation according to the manufacturer’s instructions (Sigma-Aldrich). Purified PBMCs were cultured for 5 days in MACS medium (Dulbecco’s modified Eagle’s medium containing 10% serum, 2 mM glutamine, 5 mg/ml penicillin and streptomycin). Normoxic and hypoxic C. albicans cells were fixed with 50 mM thimerosal (Sigma) and washed 4 times with sterile 1× PBS (Sigma-Aldrich). These yeast cells were incubated with PBMCs (ratio of 5:1, yeast cells/PBMCs) for 24 h, whereupon 100 µl of supernatant was collected and the cytokines and chemokines were quantified using the Luminex screening kit (R&D Systems, Abingdon, UK) in the BioPlex 200 system (Bio-Rad, Watford, UK) according to the manufacturer’s recommendations.

### Phagocytosis assays.

Bone marrow-derived macrophages (BMDMs) were prepared following extraction of bone marrow from the femurs and tibias of 12-week-old male C57BL/6 mice aged and differentiated for 7 days as described previously (105). Normoxic and hypoxic C. albicans cells were fixed with thimerosal (described above), mixed with macrophages at a ratio of 3:1 (yeast cells/macrophages), and imaged at 1-min intervals for up to 4 h using established protocols with a Nikon Eclipse Ti UltraVIEW VoX spinning disk microscope (99, 106, 107). The C. albicans cells were fixed to allow subsequent confirmation, by cytometry, that these specific hypoxic cell populations displayed β-glucan masking. The percentages of macrophages phagocytosing yeast cells and the number of yeast cells engulfed per macrophage were quantified at hourly time intervals. The difference between conditions for each time point was determined using ANOVA with Bonferroni’s *post hoc* test.

### Ethics statement.

Blood samples from healthy volunteers were collected with the informed consent of these donors and according to local guidelines and regulations that were approved by the College Ethics Review Board of the University of Aberdeen (CERB/2012/11/676).

Three 7-week-old male C57BL/6 mice were used for the preparation of BMDMs. These mice, which were selected randomly, were bred in-house, housed in stock cages under specific-pathogen-free conditions. They underwent no surgical procedures prior to culling by cervical dislocation. All animal experimentation was approved by the UK Home Office and by the University of Aberdeen Animal Welfare and Ethical Review Body.

### Statistical analyses.

Statistical analyses were performed in GraphPad Prism 5. Results from independent replicate experiments are expressed as means ± standard deviations. One-way ANOVA (Tukey’s multiple-comparison test) was used to test the statistical difference between two sets of data with a nonparametric distribution. The following *P* values were considered statistically significant and indicated as follows: *, *P < *0.05; **, *P <*0.01; ***, *P < *0.001; ****, *P < *0.0001.

10.1128/mBio.01318-18.5 MOVIE S3 Time-lapse video of BMDM interactions with hypoxic C. albicans cells. Movies S3 and S4 are representative of 12 movies (4 movies from 3 mice), that illustrate the first two hours of interactions between BMDMs and hypoxic C. albicans interactions. Download Movie S3, AVI file, 19.0 MB. Copyright © 2018 Pradhan et al. 2018 Pradhan et al. This content is distributed under the terms of the Creative Commons Attribution 4.0 International license.

10.1128/mBio.01318-18.6 MOVIE S4 Time-lapse video of BMDM interactions with hypoxic C. albicans cells. Movies S3 and S4 are representative of 12 movies (4 movies from 3 mice), that illustrate the first two hours of interactions between BMDMs and hypoxic C. albicans interactions. Download Movie S4, AVI file, 19.4 MB. Copyright © 2018 Pradhan et al. 2018 Pradhan et al. This content is distributed under the terms of the Creative Commons Attribution 4.0 International license.
