# Supplementary material for: Hypoxia Promotes Immune Evasion by Triggering β-Glucan Masking on the Candida albicans Cell Surface via Mitochondrial and cAMP-Protein Kinase A Signaling
Source: mBio. 2018 Nov 6;9(6):e01318-18. doi: 10.1128/mBio.01318-18 (PMC6222127; doi:10.1128/mBio.01318-18)
Supplement: TABLE S1 [file mbo005184148st1.pdf]

Table S1. Strains used in this study

Strain	Name	Parent	Genotype	Source
SC5314	SC5314	-	blood isolate	Gillum *et al.* (1984)
CAF2-1	CAF2-1	SC5314	*ura3Δ::imm434/URA3*	Fonzi & Irwin (1993)
CAI4	CAI4	CAF2-1	*ura3Δ::imm434/Δura3Δ::imm434*	Fonzi & Irwin (1993)
CAF4-2	CAF4-2	CAF2-1	*ura3Δ::imm434/Δura3Δ::imm434*	Fonzi & Irwin (1993)
CAI4	CAI4	SC5314	*ura3Δ::imm434/Δura3Δ::imm434*	Fonzi & Irwin (1993)
Ca372	CAI4+CIp10	CAI4	CAI4, *RPS1-CIp10 (URA3)*	Murad *et al.* (2000)
RM1000	RM1000	CAI4	*ura3Δ::imm434/Δura3Δ::imm434, his1Δ::hisG/his1Δ::hisG*	Negredo *et al.* (1997)
Ca674	RM1000+CIp20	RM1000	RIM1000, *RPS1-CIp20 (URA3,HIS1)*	Smith *et al.* (2004)
BWP17	BWP17	RM1000	*ura3Δ::imm434/Δura3Δ::imm434, his1Δ::hisG/his1Δ::hisG, arg4Δ::hisG/arg4Δ::hisG*	Wilson *et al.* (1999)
Ca1206	BWP17+CIp30	RM1000	BWP17, *RPS1-CIp30 (URA3,HIS1,ARG4)*	Dennison *et al.* (2005)
DAY185	DAY185	BWP17	BWP17, *his1::hisG::HIS1*	Davis *et al.* (2000)
SN95	SN152	CAF2-1	*arg4Δ/arg4Δ, his1Δ/his1Δ, URA3/ura3Δ::imm434, IRO1/iro1Δ::imm434*	Noble & Johnson (2005)
SN148	SN148	CAF2-1	*arg4Δ/arg4Δ, leu2Δ/leu2Δ, his1Δ/his1Δ, ura3Δ::imm434/ura3Δ::imm434, iro1Δ::imm434/iro1Δ::imm434*	Noble & Johnson (2005)
SN152	SN152	CAF2-1	*arg4Δ/arg4Δ, leu2Δ/leu2Δ, his1Δ/his1Δ, URA3/ura3Δ::imm434, IRO1/iro1Δ::imm434*	Noble & Johnson (2005)
LR2	*gpr1Δ*	CAI4	CAI4, *gpr1 ::hisG/gpr1 ::hisG-URA3-hisG*	Maidan *et al.* (2005)
NM6	*gpa2Δ*	CAI4	CAI4, *gpa2 ::hisG/gpa2 ::hisG-URA3-hisG*	Maidan *et al.* (2005)
NM23	*gpr1Δ gpa2Δ*	CAI4	CAI4, *gpa2Δ::hisG/gpa2Δ::hisG, gpr1Δ::hisG/gpr1Δ::hisG-URA3-hisG*	Maidan *et al.* (2005)
DSY2195	*crz1Δ*	CAF4-2	CAF4-2, *crz1Δ::hisG/crz1Δ::hisG::URA3::hisG*	Karababa *et al.* (2006)
HLC52	*efg1Δ*	CAI4	CAI4, *efg1::hisG/efg1::hisG-URA3-hisG*	Lo *et al.* (1997)
JKC19	*cph1Δ*	CAI4	CAI4, *cph1::hisG/cph1::hisG-URA3-hisG*	Liu *et al.* (1994)
CaAS18	*tec1Δ*	CAI4	CAI4, *tec1Δ::hisG/ tec1Δ::hisG, pVEC-URA3*	Schweizer *et al.* (2000)
CJN702	*bcr1Δ*	DAY185	DAY185, *bcr1Δ::ARG4/bcr1Δ::URA3*	Nobile & Mitchell (2005)
CaLC700	*mkc1Δ*	SN95	SN95, *mkc1Δ::FRT/mkc1Δ::FRT*	LaFayette *et al.*, (2010)
JC482	*ssk2Δ*	BWP17	BWP17, *ssk2Δ::loxP-ARG4-loxP/ssk2Δ::loxP-HIS1-loxP*	Cheetham *et al.* (2007)
JC74	*pbs2Δ*	BWP17	BWP17, *pbs2Δ::loxP-ARG4-loxP/pbs2Δ::loxP-HIS1-loxP*	Cheetham *et al.* (2007)
JC50	*hog1Δ*	CAI4	CAI4, *hog1Δ::loxP-ura3-loxP/hog1Δ::loxP-HIS1-LoxP, CIp20-URA3, HIS1*	Smith *et al.* (2004)
CR323	*cyr1Δ* (*cdc35Δ*)	CAI4	CAI4, *cdc35Δ::hisG/cdc35Δ::hisG, pVEC-URA3*	Rocha *et al.* (2001)
tpk1Δ	*tpk1Δ*	SN152	SN152, *tpk1::HIS1/ tpk1::ARG4*	Cao *et al.* (2017)
tpk2Δ	*tpk2Δ*	SN152	SN152, *tpk2::HIS1/ tpk2::ARG4*	Cao *et al.* (2017)
tpk1Δ tpk2Δ	*tpk1Δ tpk2Δ*	SN152	SN152, *tpk1::LEU2/ tpk1::FRT, tpk2::HIS1/ tpk2::ARG4*	Cao *et al.* (2017)
YCAT39	*ccr4Δ*	DAY185	DAY185, *ccr4Δ::ARG4/ccr4Δ::URA3*	Dagley *et al.* (2011)
YCAT51	*pop2Δ*	DAY185	DAY185, *pop2Δ::ARG4/pop2Δ::URA3*	Dagley *et al.* (2011)

WH324	*aox1Δ*	CAI4	CAI4, *aox1aΔ::hisG/aox1bΔ::hisG*	Kuh & Kang (2001)
GOA31	*goa1Δ*	SN148	SN148, *goa1Δ::URA3/goa1Δ::ARG4 arg4/arg4leu2/leu2 his1/his1ura3::imm434/ura3::imm434 iro1::imm434*	Bambach *et al.* (2009)
UPC2M4A	*upc2Δ*	SC5314	SC5314, *upc2-1Δ::FRT/upc2-2Δ::FRT*	Vasicek *et al.* (2014)
CA-IF003	*sod1Δ*	SN152	SN152*, sod1Δ::cmLEU2/sod1Δ::CdHIS1*	Frohner *et al.* (2009)
CA-IF007	*sod2Δ*	SN152	SN152, *sod2Δ::cmLEU2/sod2Δ::CdHIS1*	Frohner *et al.* (2009)
CA-IF011	*sod3Δ*	SN152	SN152, *sod3Δ::cmLEU2/sod3Δ::CdHIS1*	Frohner *et al.* (2009)
CA-IF070	*sod4/5/6Δ*	SN152	SN152, *sod5Δ::cmLEU1/sod5Δ::Cd HIS1, sod4Δ::FRT/sod4Δ::FRT, sod6Δ::FRT/sod6Δ::FRT*	Frohner *et al.* (2009)

CLINICAL ISOLATES

**Isolate**	**Clade**	**Information**	**Source**
C. albicans			
SC5314	1	Blood	Gillum *et al.* (1984)
IHEM16614	2	Oropharynx	MacCallum *et al* (2009)
J990102	3	Vagina	MacCallum *et al* (2009)
AM2005/0377	4	Blood	MacCallum *et al* (2009)
C. auris			
NCPF 8980 #9		South African	Dr. Liz Johnson, Public Health England
NCPF 8984 #15		Japanese/Korean	Dr. Liz Johnson, Public Health England
NCPF 8985 #20		South Asian/India	Dr. Liz Johnson, Public Health England
C. glabrata			
SCS130399L		Blood	Odds *et al.* (2007)
SCS123636D		Blood	Odds *et al.* (2007)
AM2004/0091		Central line	
C. tropicalis			
SCS122443V		Blood	Odds *et al.* (2007)
SCS76638K		Blood	Odds *et al.* (2007)
b30357/6/04		IV catheter	
C. parapsilosis			
SCSXM70052		Blood	Odds *et al.* (2007)
CSBB425167		Blood	Odds *et al.* (2007)
SCS73972P		Blood	Odds *et al.* (2007)
C. krusei			
SCS71987M		Blood	Odds *et al.* (2007)

EJ30846/01/04		Femoral line tip	Odds *et al.* (2007)
C. guilliermondii			
SCSBB418097		Blood	Odds *et al.* (2007)
SCS74937K		Blood	Odds *et al.* (2007)
SCSMB042615		Blood	Odds *et al.* (2007)
S. cerevisiae			
S288c		Laboratory strain	ATCC
40V		Blood	Donna MacCallum
J940421P		Blood	Donna MacCallum
NCPF8313		Blood	Dr Elizabeth M. Johnson

References

Bambach *et al.* (2009) Goa1p of *Candida albicans* localizes to the mitochondria during stress and is required for mitochondrial function and virulence. *Eukaryotic Cell* 8, 1706–1720.

Cao C *et al.* (2017) Global regulatory roles of the cAMP/PKA pathway revealed by phenotypic, transcriptomic and phosphoproteomic analyses in a null mutant of the PKA catalytic subunit in *Candida albicans*. *Molec Microbiol* 105, 46-64.

Chauvel M *et al.* (2012) A versatile overexpression strategy in the pathogenic yeast Candida albicans: identification of regulators of morphogenesis and fitness. *PLoS ONE* 7, e45912.

Cheetham J *et al*. (2007) A single MAPKKK regulates the Hog1 MAPK pathway in the pathogenic fungus *Candida albicans*. *Molec. Biol. Cell.* 18**,** 4603-4614.

Dagley MJ *et al.* (2011) Cell wall integrity is linked to mitochondria and phospholipid homeostasis in *Candida albicans* through the activity of the post-transcriptional regulator Ccr4-Pop2. *Molec Microbiol* 79, 968–989.

Davis D *et al.* (2000) *Candida albicans RIM101* pH response pathway is required for host-pathogen interactions. *Infection Immunity* 68, 5953–5959.

Dennison PM *et al*. (2005) Gene disruption in *Candida albicans* using a synthetic, codon-optimised Cre-loxP system. *Fungal Genet. Biol.* 42, 737-748.

Fonzi WA and Irwin MY (1993) Isogenic strain construction and gene mapping in Candida albicans. *Genetics* 134, 717-728.

Frohner IE *et al*. (2009) Candida albicans cell surface superoxide dismutases degrade host-derived reactive oxygen species to escape innate immune surveillance. *Molec. Microbiol*. 71**,** 240–252.

Gillum AM *et al.* (1984) Isolation of the *Candida albicans* gene for orotidine-5'-phosphate decarboxylase by complementation of *S. cerevisiae ura3* and *E. coli pyrF* mutations. *Molec. Gen. Genet*. 198, 179-182.

Huh WK and Kang SO (2001) Characterization of the gene family encoding alternative oxidase from *Candida albicans. Biochem. J.* 356, 595–604.

Karababa M *et al.* (2006) CRZ1, a target of the calcineurin pathway in Candida albicans. *Molec Microbiol*. 59, 1429-1451.

LaFayette SL *et al.* (2010) PKC signaling regulates drug resistance of the fungal pathogen Candida albicans via circuitry comprised of Mkc1, calcineurin, and Hsp90. *PLoS Pathog* 6, e1001069.

Liu H *et al*. (1994) Suppression of hyphal formation in *Candida albicans* by mutation of a *STE12* homolog. *Science* 266, 1723-1726.

Lo HJ *et al.* (1997) Nonfilamentous *C. albicans* mutants are avirulent. *Cell* 90, 939-949.

Maidan MM et al. (2005) The G protein-coupled receptor Gpr1 and the Galpha protein Gpa2 act through the cAMP-protein kinase A pathway to induce morphogenesis in Candida albicans. *Molec Biol Cell*. 16, 971-1986.

MacCallum *et al.* (2009) Property differences among the four major *Candida albicans* strain clades. *Eukaryotic Cell* 8, 373–387.

Murad, AMA *et al.* (2000) CIp10, an efficient and convenient integrating vector for *Candida albicans*. *Yeast,* 16, 325-327

Negredo A *et al.* (1997) Cloning, analysis and one-step disruption of the ARG5,6 gene of Candida albicans. *Microbiology* 143, 297-302.

Nobile CJ and Mitchell AP (2005) Regulation of cell-surface genes and biofilm formation by the *C. albicans* transcription factor Bcr1p. *Curr. Biol.* 15, 1150–1155.

Noble SM and Johnson AD (2005) Strains and strategies for large-scale gene deletion studies of the diploid human fungal pathogen Candida albicans. *Eukaryot Cell*. 4, 298-309.

Odds FC *et al.* (2007) One year prospective survey of *Candida* bloodstream infections in Scotland. *J Med Microbiol* 56**,** 1066–1075.

Rocha CRC *et al*. (2001) Signaling through adenylyl cyclase is essential for hyphal growth and virulence in the pathogenic fungus *Candida albicans*. *Molec. Biol. Cell* 12, 3631-3643.

Schweizer A *et al*. (2000) The TEA/ATTS transcription factor CaTec1p regulates hyphal development and virulence in *Candida albicans*. *Molec. Microbiol.* 38, 435-445.

Smith DA *et al.* (2004) A conserved stress-activated protein kinase regulates a core stress response in the human pathogen *Candida albicans*. *Molec. Biol. Cell* 15, 4179-4190.

Vasicek EM *et al.* (2014) *UPC2* is universally essential for azole antifungal resistance in *Candida albicans. Eukaryotic Cell* 13. 7933-7946.

Wilson RB *et al.* (1999) Rapid hypothesis testing with Candida albicans through gene disruption with short homology regions. *J Bacteriol* 181, 1868-1874.
