# Supplementary material for: Hypoxia Promotes Immune Evasion by Triggering β-Glucan Masking on the Candida albicans Cell Surface via Mitochondrial and cAMP-Protein Kinase A Signaling
Source: mBio. 2018 Nov 6;9(6):e01318-18. doi: 10.1128/mBio.01318-18 (PMC6222127; doi:10.1128/mBio.01318-18)
Supplement: FIG S1 [file mbo005184148sf1.pdf]

Figure S1

A

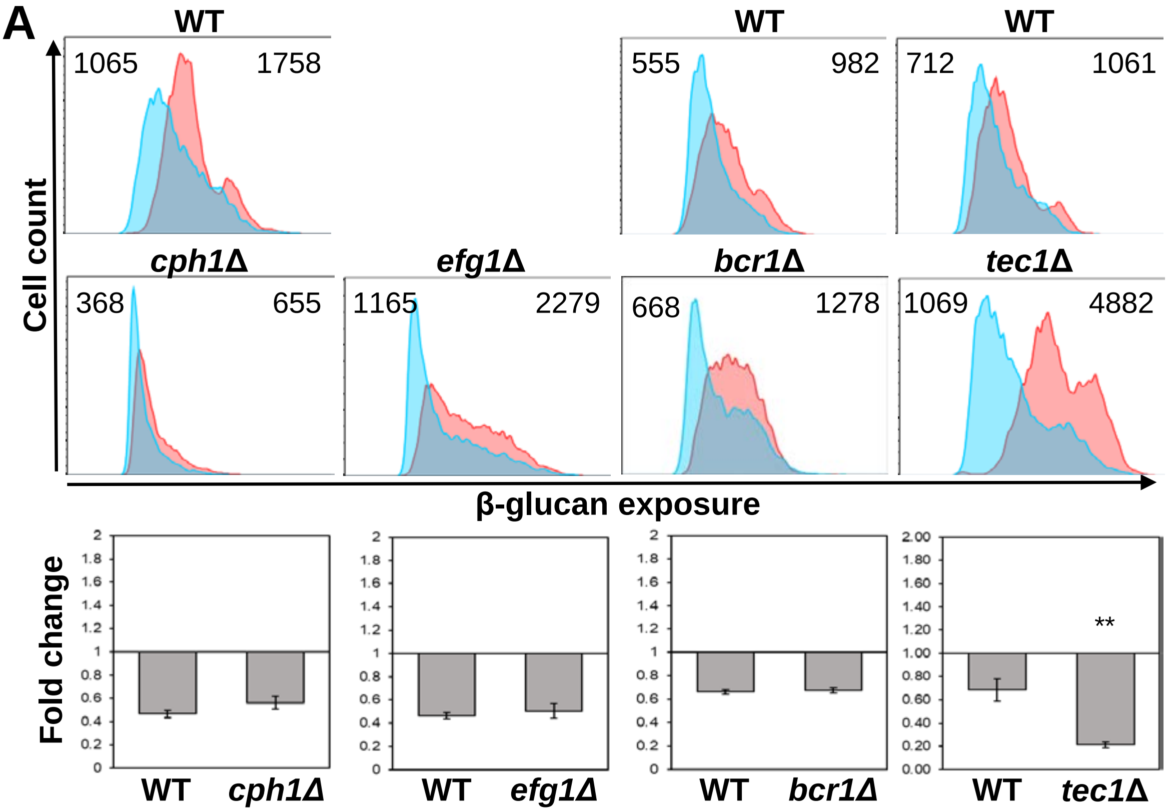

B

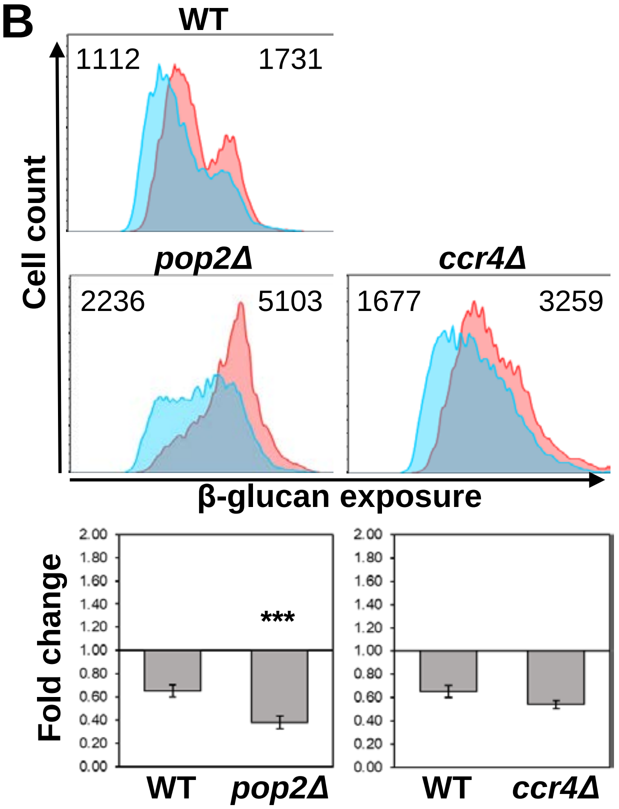

Figure S1. Hypoxia-induced β-glucan masking in specific *C. albicans* mutants. Analysis of β-glucan masking by *C. albicans* mutants by Fc-dectin-1 staining of cells grown under normoxic (pink) or hypoxic conditions (cyan) (upper panels). Median Fluorescence Intensities (MFIs) for hypoxic and normoxic cell populations are shown. The corresponding wild type control is shown above each mutant or set of mutants. Fold changes in β-glucan exposure (lower panels) represent the MFI under hypoxic conditions divided by the MFI for the corresponding control normoxic cells. Means and standard deviations from three independent replicate experiments are shown, and the data were analysed using ANOVA with Tukey's multiple comparison test: *, $p \leq 0.05$; **, $p \leq 0.01$; ***, $p \leq 0.001$. **(A)** Morphogenetic mutants: WT, wild type, SC5314; *cph1Δ,* JKC19; *efg1Δ*, HLC52; *bcr1Δ,* CNJ702; *tec1Δ*, CaAS18 (Table S1). **(B)** Mutants affecting mitochondrial functionality: WT, wild type, DAY152; *pop2Δ,* YCAT51; *ccr4Δ,* YCAT39. The data for the *efg1Δ*, *tec1Δ* and *ccr4Δ* mutants also appear in Figures 4 and 6.
